# *Drosophila* Tet Is Expressed in Midline Glia and Is Required for Proper Axonal Development

**DOI:** 10.3389/fncel.2019.00252

**Published:** 2019-06-04

**Authors:** Joy N. Ismail, Shireen Badini, Felice Frey, Wassim Abou-Kheir, Margret Shirinian

**Affiliations:** ^1^Department of Experimental Pathology and Immunology, Faculty of Medicine, American University of Beirut, Beirut, Lebanon; ^2^Center for Infectious Diseases Research, American University of Beirut Medical Center, Beirut, Lebanon; ^3^Department of Anatomy, Cell Biology, and Physiological Sciences, Faculty of Medicine, American University of Beirut, Beirut, Lebanon

**Keywords:** Tet, *Drosophila*, midline glia, neurodevelopment, axon guidance cue, axon guidance defect

## Abstract

Ten-Eleven Translocation (TET) proteins are important epigenetic regulators that play a key role in development and are frequently deregulated in cancer. *Drosophila melanogaster* has a single homologous Tet gene (*dTet*) that is highly expressed in the central nervous system during development. Here, we examined the expression pattern of dTet in the third instar larval CNS and discovered its presence in a specific set of glia cells: midline glia (MG). Moreover, dTet knockdown resulted in significant lethality, locomotor dysfunction, and alterations in axon patterning in the larval ventral nerve cord. Molecular analyses on *dTet* knockdown larvae showed a downregulation in genes involved in axon guidance and reduced expression of the axon guidance cue Slit. Our findings point toward a potential role for dTet in midline glial function, specifically the regulation of axon patterning during neurodevelopment.

## Introduction

Epigenetic regulation of gene expression is essential for proper development of the central nervous system. In mammals, the Ten-Eleven Translocation proteins (TET1-3) are a family of methylcytosine dioxygenases that catalyze the conversion of 5-methylcytosine (5mC) into 5-hydroxylmethylcytosine (5hmC) and further oxidized derivatives. Thymine DNA Glycosylase (TDG) then excises the oxidized base, leading to the restoration of an unmodified cytosine residue. TET proteins were initially discovered when TET2 was found to be a part of a translocation protein with Mixed Lineage Leukemia (MLL) in a subset of patients with Acute Myeloid Leukemia (AML) (Ono et al., 2002; Lorsbach et al., 2003). Over the last decade, TET proteins have been found to be dysregulated in various types of cancers including solid tumors (Kosmider et al., 2009; Kraus et al., 2015). They are expressed throughout development and may play a role in proliferation and differentiation processes (reviewed in Rasmussen and Helin, 2016). While each TET protein appears to be important in specific tissues at particular stages during development, there is some degree of redundancy in the function of each protein. For instance, mice with *Tet1* or *Tet2* knockout show far less severe phenotypes than double knockout mice (Dawlaty et al., 2013). On the other hand, *Tet3* knockout led to neonatal lethality in mice, indicating that *Tet1* and *Tet2* cannot compensate for this loss and pointing toward the critical role of *Tet3* in early development (Gu et al., 2011). Interestingly, the highest concentration of 5hmC is found in the brain (Globisch et al., 2010; Song et al., 2011). Similarly, TET proteins were found to be highly expressed in the mammalian brain, especially within the cortex and hippocampus (Mi et al., 2015).

Tet proteins are also expressed in invertebrates, indicating their evolutionary conservation. *Drosophila melanogaster* possesses one Tet gene (*dTet*) located on Chromosome 3 (62F6-63A1) that encodes six transcripts. The catalytic domain of dTet proteins is most similar in structure to mammalian TET3 (Dunwell et al., 2013). Although dTet is capable of converting DNA 5mC into 5hmC *in vitro*, 5hmC on DNA was estimated to comprise only around 100 bases in the fly genome (Zhang et al., 2015). Recently, dTet was reported to demethylate 5mC on RNA (5mrC) (Delatte et al., 2016) and demethylate 6-methyladenine (6mA) on DNA (Zhang et al., 2015).

The development of more sensitive techniques for the detection of base modifications led to the rejection of the previous notion that methylation is absent from the fly genome (reviewed in Dunwell and Pfeifer, 2014). However, the presence of methylation was regarded skeptically since flies lack homologs of DNA methyltransferases (DNMTs) 1 and 3, which are needed for maintenance or *de novo* methylation, respectively. Rather, they express a homolog of DNMT2, Mt2, which has been shown to methylate tRNA molecules (Kunert et al., 2003; Phalke et al., 2009). One possible explanation may be the presence of a methyltransferase enzyme that has not been discovered yet (Takayama et al., 2014) and the presence of a demethylating enzyme (dTet) further strengthened this possibility (Zhang et al., 2015).

In humans, the 5hmrC mark is most prevalent in mRNA molecules (Huang et al., 2016). In mice, similar to the 5hmC mark, 5hmrC was found to be expressed in brain tissue (Miao et al., 2016). 6mA, a mark that was initially identified in the prokaryotic genome (Vanyushin et al., 1968), was shown to be present in lower eukaryotes and then mammals and may be important for development (Fu et al., 2015; Huang et al., 2015; Liu et al., 2016). Furthermore, 6mA appears to be sensitive to environment as it increased in the brains of mice upon exposure to stress (Yao et al., 2018).

The presence of 6mA or 5mrC is associated with reduced gene expression (Delatte et al., 2016; Xie et al., 2018). Interestingly, both modifications were shown to be present at higher levels in glioblastoma patients, thus pointing toward its relevance to disease (Kraus et al., 2015; Xie et al., 2018). It is important to note that expression of TET proteins is dysregulated in glioblastoma tissue and cell samples (Orr et al., 2012; Takai et al., 2014). Taken together, it appears that the catalytic function of TET proteins is essential for protecting against the consequences of excessive 6mA or 5mrC at tumor suppressor genes (Esteller and Herman, 2002; Xie et al., 2018).

Notably, in an analysis of RNA sequencing data from different fly tissues throughout all developmental stages, dTet expression was found to be highest in the brain, peaking at the third instar larval stage (Dunwell et al., 2013). The larval brain contains many specialized cell populations that are necessary for developmental processes such as neuroblasts, ganglion mother cells, and midline glia (MG) in the ventral nerve cord (VNC). Midline glial cells are a subclass of neuropil glia that are only expressed in the developing fly and are eliminated during the pupal stage prior to adult eclosion (Awad and Truman, 1997). The *Drosophila* MG and mammalian floorplate cells are morphologically and functionally similar (Crews, 2010). MG play a major role in regulating axon connectivity in the ventral nerve cord, a process that is dependent on their ability to synthesize and secrete attractive and repulsive molecules, namely Netrins and Slit, respectively (Noordermeer et al., 1998).

Recent studies have shown that dTet knockout leads to lethality and locomotor phenotypes (Zhang et al., 2015; Wang et al., 2018). In addition, dTet was reported to participate in numerous neuronal functions such as the maintenance of circadian rhythm and regulating the expression of genes involved in neuronal differentiation (Wang et al., 2018; Yao et al., 2018). Although dTet expression peaks at the larval stage, its function and presence in the larval brain is still not fully understood. In order to investigate the role of dTet in brain development, we sought to identify the specific cell populations in which dTet is expressed. Here, we report that dTet is expressed in larval brain neurons as described in Wang et al. (2018), however, we also identify a prominent expression of dTet in MG cells in the larval VNC. Considering the importance of MG in axonal guidance, we used RNA interference (RNAi) to knockdown dTet and subsequently analyzed the effects on axon patterning. Interestingly, we detected defects in axon commissures in the ventral nerve cord that potentially contribute to the observed locomotor phenotype.

## Materials and Methods

### Fly Stocks

Flies were maintained on standard cornmeal-agar medium at 29°C unless otherwise indicated. dTet-GFP flies were kindly provided by Ruth Steward. Tubulin-Gal4 (#5138), Slit-Gal4 (#9580), and Sim-Gal4 (#9150) fly stocks were obtained from Bloomington Drosophila Stock Center. dTet-RNAi (#102273 and #36187) and mCherry-RNAi (#35785) flies were obtained from Vienna Drosophila RNAi Center.

### Quantitative RT-PCR and Sequencing

Total RNA was extracted from 15 third instar larvae using TRI Reagent (Sigma-Aldrich). cDNA synthesis was then performed using the RevertAid First Strand cDNA Synthesis Kit (ThermoScientific). Quantitative RT-PCR reactions were performed in triplicates on Biorad CFX Connect using SYBR Green (BioRad SSO Advanced Universal SYBR Green Supermix). All samples were normalized to *Rp49* and gene expression relative to control was calculated using the ΔΔCt method. For sequencing, genomic DNA was extracted from 10 adult flies, amplified by PCR, and then sequenced using EGFP primers (Venken et al., 2011). Primer sequences are listed in the Supplementary Table 1.

### Locomotor Analysis

Wandering third instar larvae were collected and placed individually on a 2% agarose plate on top of a 0.5 cm^2^ grid. Once the larva made its first movement, the number of lines that it crossed in 1 min was recorded (adapted from Nichols et al., 2012). Fifty larvae were scored per genotype. A two-tailed Student’s *t*-test was used to determine significance between groups.

### Immunofluorescence Staining

Embryos were collected from grape juice plates, placed in 50% bleach in distilled water for 2 min, and then fixed in a 1:1 solution of 4% formaldehyde in PBS and n-heptane. Embryos were subsequently devitinelized in methanol and washed with methanol prior to staining as indicated below. Third instar larval brains were dissected in PBS with 0.3% Triton X-100 (PBST) and were then fixed in 4% formaldehyde for 20 min at room temperature. Brains were then washed in PBST three times for 20 min each. Next, brains were placed in blocking solution [5% normal goat serum (Dako) in PBST] overnight at 4°C. Subsequently, samples were incubated in primary antibody diluted in blocking solution overnight at 4°C. The following antibodies were used: rabbit anti-GFP (Abcam, 1:2000), mouse anti-Wrapper [10D3, Developmental Studies Hybridoma Bank (DSHB), 1:20], mouse anti-Slit (DSHB, C555.6D, 1:50), mouse anti-FasIII (7G10, DSHB, 1:30), mouse anti-Prospero (DSHB, MR1A, 1:100). The brains were then washed in PBST three times for 20 min each and incubated with fluorochrome-conjugated secondary antibodies AlexaFluor-488 anti-rabbit or AlexaFluor-594 anti-mouse (Abcam, 1:500) for 2 h at room temperature. Next brains were incubated in DAPI solution (1:5000, 10^−3^ mg/mL, Molecular Probes) for 5 min and washed in PBST three times for 20 min each. Finally, samples were mounted onto microscope slides with gold anti-fade solution (Invitrogen) for subsequent analysis using the Zeiss LSM 710 laser scanning confocal microscope. All images were acquired and analyzed using the Zeiss ZEN 9 imaging software.

### Analysis of Axonal Defects

Confocal images were randomly shuffled and scored by two blinded individuals. Thirty brains were analyzed per genotype. Scoring was based on counting the number of segments at which axons diverge away from the midline (adapted from Mosca and Schwarz, 2010).

### Survival Analysis

Twenty third instar larvae were collected from each cross and placed into new vials at 29°C. Subsequently, the number of adults that eclosed was recorded. The survival assay was performed three times and a two-tailed Student’s *t*-test was used to determine significance between groups.

### Western Blot

Thirty-five larval brains were dissected in PBS and collected in 2× Laemmli buffer containing 4% protease inhibitor (Roche) and 10% phosphatase inhibitor (Roche). Samples were homogenized using a pestle followed by sonication for 10 min at 4°C. Next, samples were centrifuged for 15 min and the supernatant was collected. The protein concentration was measured using a Nanodrop Spectrophotometer. 100 μg of protein per lane was loaded onto 8% SDS gel and run at 90V. Precision Plus Protein Kaleidoscope ladder (Biorad) was used as a molecular weight marker. Blotting was done overnight at 30V at 4°C using a PVDF membrane. The membrane was then placed in blocking solution (5% milk in PBS-Tween 0.05%) for 1 h. The primary antibody was added overnight in blocking solution (anti-Slit, DSHB, 1:200; anti-βactin, Abcam, 1:5000). The membrane was washed three times in PBS-Tween for 10 min each and incubated with goat anti-rabbit or anti-mouse HRP (Santa Cruz, 1:5000) for 2 h at room temperature. The membrane was washed three times with PBS-Tween for 10 min each. Imaging was done on the Chemidoc MP machine using ECL Clarity Max (Biorad). Quantification of relative expression was performed on ImageJ by normalizing signal intensity of each band to that of its loading control. The average of three replicates was calculated and a two-tailed Student’s *t*-test was used to determine significance relative to controls.

### Dot Blot

DNA or RNA was extracted using 50 or 30 brains, respectively. Samples were dotted onto a nylon membrane. The membrane was then stained with methylene blue for visualization of loading. The membrane was washed with distilled water and was subsequently blocked in 5% milk in PBS-Tween (0.05%) for 1 h at room temperature. The membrane was then incubated with the primary antibody – rabbit anti-6mA for DNA (Synaptic Systems, 1:1000) and anti-5hmC for RNA (Abcam, 1:5000) – overnight at 4°C. The following day the membrane was washed three times with PBS-Tween and was subsequently incubated with a rabbit HRP-conjugated secondary antibody (Santa Cruz, 1:5000) for 2 h at room temperature. The membrane was washed three times and was then imaged using a ChemiDoc. Relative quantification and normalization to the loading control was performed using ImageJ.

## Results

### *dTet* Is Expressed in Midline Glial Cells

**FIGURE 1:**
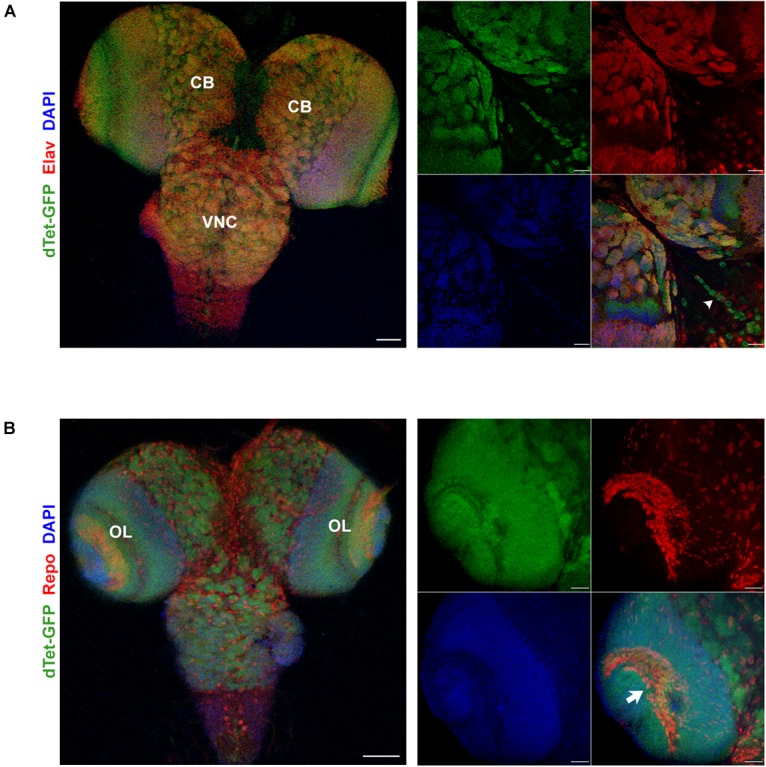
dTet is expressed in neurons and a subset of glia in the third instar larval CNS. **(A)** Left, maximum intensity projection at low magnification showing overlap between dTet-GFP in Elav-positive neurons. Scale bar, 50 μm. Right, high magnification micrograph of neurons in central brain co-expressing Elav and dTet-GFP. Arrowhead indicating midline glia pattern. Scale bar, 20 μm. **(B)** Left, maximum intensity projection at low magnification showing expression of dTet-GFP and Repo-positive glial cells. Scale bar, 50 μm. Right, high magnification maximum intensity projection showing expression of dTet-GFP within glia in the optic lobe (arrow). Scale bar, 20 μm. CB, central brain, VNC, ventral nerve cord, OL, optic lobe.

**FIGURE 2:**
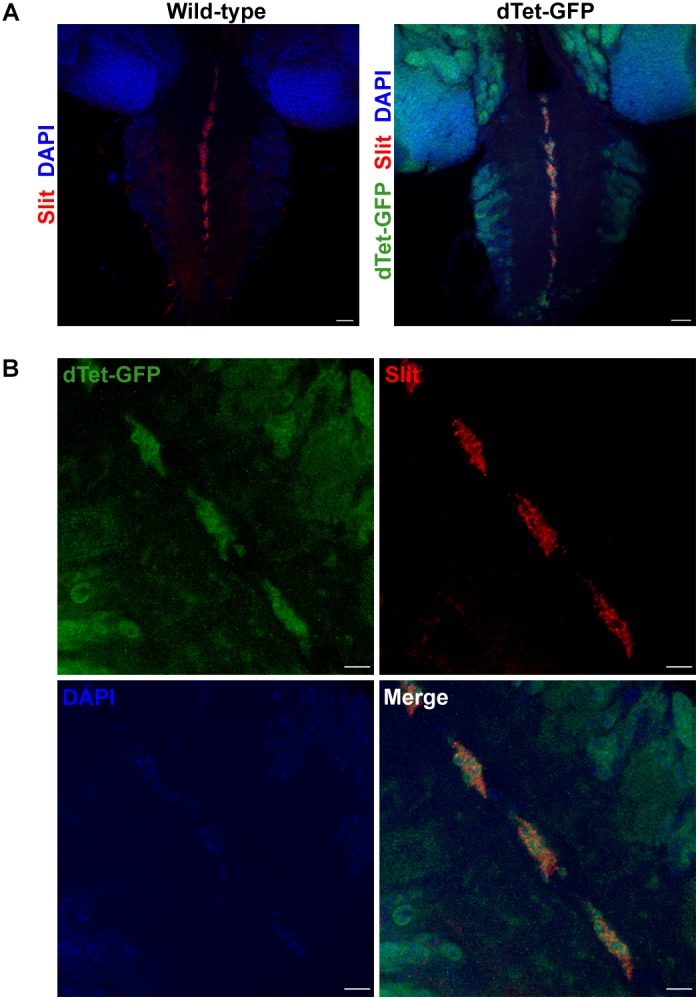
dTet is present in midline glial cells in the ventral nerve cord of third instar larval CNS. **(A)** In wild-type controls, anti-Slit labels midline glial cells in the ventral nerve cord. In dTet-GFP flies, dTet, and Slit are co-expressed. Scale bar, 50 μm. **(B)** High magnification confocal micrograph showing the presence of dTet in midline glia. Scale bar, 10 μm.

To characterize the expression pattern of dTet in the larval brain, we used flies with a MiMIC-mediated GFP insertion within an intronic site in the *dTet* gene (Delatte et al., 2016). This results in the expression of a functional dTet-GFP fusion protein that is under the control of the endogenous dTet promoter. Quantitative real-time PCR (qRT-PCR) confirmed that dTet-GFP flies express dTet at comparable levels to wild-type control flies (Supplementary Figure 1). Furthermore, sequencing of genomic DNA from these flies confirmed the presence of EGFP within the dTet locus (Supplementary Table 1). Previously, the highest level of dTet expression in fly embryos was detected in neurons (Wang et al., 2018). In line with that, our analysis of third instar larval brains showed extensive dTet expression in neurons in the central brain and VNC (Figure 1A). While dTet expression was low in most glial cell subtypes, we detected its expression in laminal glia within the optic lobe (Figure 1B) as well as in MG in the VNC. The MG secrete Slit, a repulsive signal that is required for preventing excessive crossing of axons across the midline. The loss of Slit leads to fusion and collapse of axon tracts into the midline (Battye et al., 1999). Next we confirmed MG specific expression of dTet by co-staining dTet-GFP brains with the MG markers Slit (Figure 2A,B) and Wrapper (Supplementary Figure 2). This was further confirmed by acquiring orthogonal projections in which dTet appeared to be specifically within the Wrapper-positive midline glia (Supplementary Figure 2). In addition, dTet is present in midline glia as early as the embryonic stage (Supplementary Figure 2).

### *dTet* Knockdown Is Associated With Survival and Locomotor Defects

**FIGURE 3:**
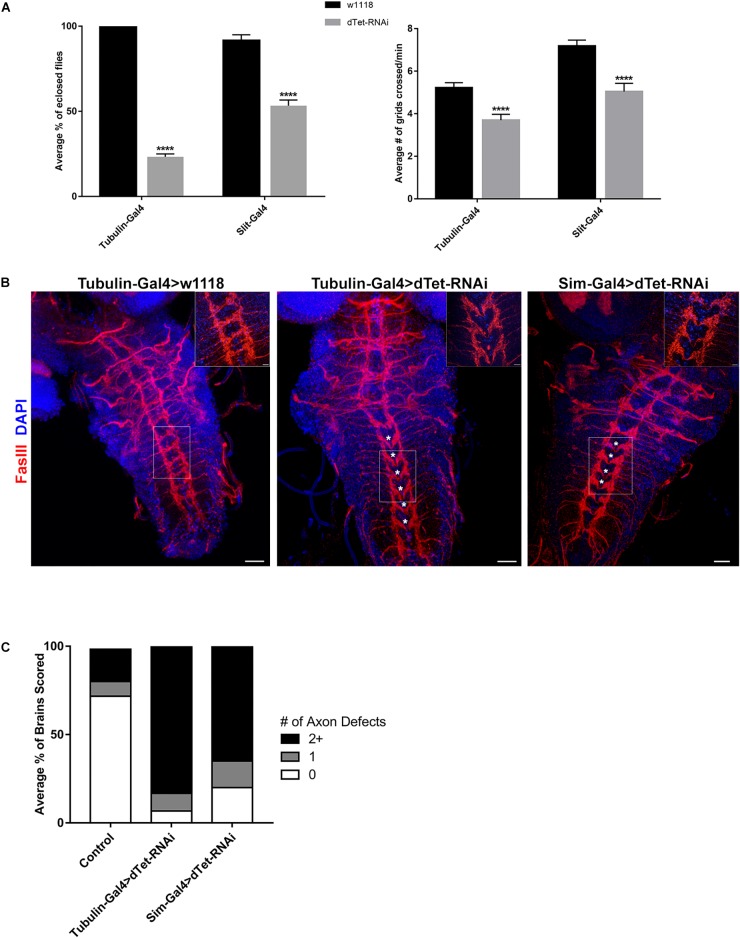
dTet knockdown affects locomotor behavior and leads to reduced survival. **(A)** Left, survival assay on third instar larvae. dTet knockdown larvae show reduced survival rates in ubiquitous (Tubulin-Gal4) and midline glial (Slit-Gal4) knockdown (*p* < 0.0001, *n* = 3, 20 larvae per group). Right, crawling assay on third instar larvae. *dTet* knockdown larvae exhibit reduced locomotor performance in both ubiquitous and midline glia-specific groups as compared to controls (*p* < 0.0001, *n* = 50). Control is Tubulin-Gal4 > w1118. Mean is shown with SEM. **(B)** Maximum intensity projections of larval VNCs with axon commissures labeled with FasIII. Asterisks indicate defects in commissure crossing. Scale bar, 20 μm. Inset, 20 μm. **(C)** Quantification of axon defects in control and knockdown VNCs (*n* = 30) where 0, 1, and 2+ errors are indicated.

We then performed ubiquitous *dTet* knockdown using Gal4/RNAi under the tubulin promoter. Only 25% of dTet knockdown larvae survived until the adult stage with the highest lethality observed at pupal stage (Figure 3A). To understand whether the lethality observed is correlated with the presence of dTet in MG, we performed knockdown of *dTet* in these specific MG cells using the Slit-Gal4 driver. dTet depletion in MG led to significantly reduced survival rates compared to controls, with approximately 50% of larvae eclosing as adults (Figure 3A). A recent study has shown that dTet mutants exhibit locomotor defects (Wang et al., 2018), hence to determine whether dTet expression in MG is correlated with locomotor function, we performed crawling assays on larvae with ubiquitous or MG specific dTet knockdown. It is important to note that because locomotion is a complex function that is based on an interaction with the environment and endogenous circuits, variability in the performance scores of larvae within the controls is expected (Günther et al., 2016). Interestingly, larvae with dTet knockdown in MG showed a decrease in locomotor performance equivalent to that seen in larvae with ubiquitous knockdown relative to controls (∼30%) (Figure 3B). This indicates that the locomotor defects observed in dTet knockdown larvae may be associated with MG function.

### *dTet* Knockdown Is Associated With Defects in Axon Patterning

Since the MG are required for axon guidance, we analyzed the midline commissures in brains stained with Fasciclin III (FasIII), an adhesion molecule expressed in the membrane of developing growth cones and axon fascicles (Snow et al., 1989). Intriguingly, we found the stereotypical meticulous organization of axons and midline commissures in the VNC disrupted in dTet-RNAi brains (Figure 3B). While axons directly cross the midline at each segment in the wild-type control, axons divert away from the midline at multiple segments and direct horizontal commissures are seen less frequently in dTet knockdown brains. 83% of knockdown brains had two or more discontinuous segments whereas this was only seen in 27% of control brains (Figure 3B,C). An additional off-target control, mCherry-RNAi, was used and 33% of VNCs showed defects at two or more segments (Supplementary Figure 3). Interestingly, the phenotype appeared to be an “all-or-nothing” phenomenon as the brains with defects typically showed 4–5 discontinuous segments whereas only few brains had just one error (Figure 3B,C). To determine whether this phenotype was specific to dTet knockdown, we utilized a second dTet-RNAi line that also showed the commissure phenotype with lesser severity (61%, Supplementary Figure 3). Due to the expression of Slit in secondary tissues such as the heart (MacMullin and Jacobs, 2006), we performed dTet knockdown using Sim-Gal4 that is specific to midline glia. The axon phenotype was present in brains of larvae with MG-specific dTet knockdown, with around 65% showing two or more defects, indicating that dTet in MG contributes to axon commissure formation (Figure 3B,C). In a study on Importin – a protein required for transporting molecules into the nucleus – Mosca and Schwarz described a similar axon phenotype in Importin mutant larval brains in addition to altered muscle patterning. Interestingly, Importin was also found to be expressed in the midline highlighting the potential link between MG and proper commissure formation in the larval brain.

### *dTet* Knockdown Leads to Downregulation of Genes Involved in Axon Guidance and Reduced Expression of Slit Protein

**FIGURE 4:**
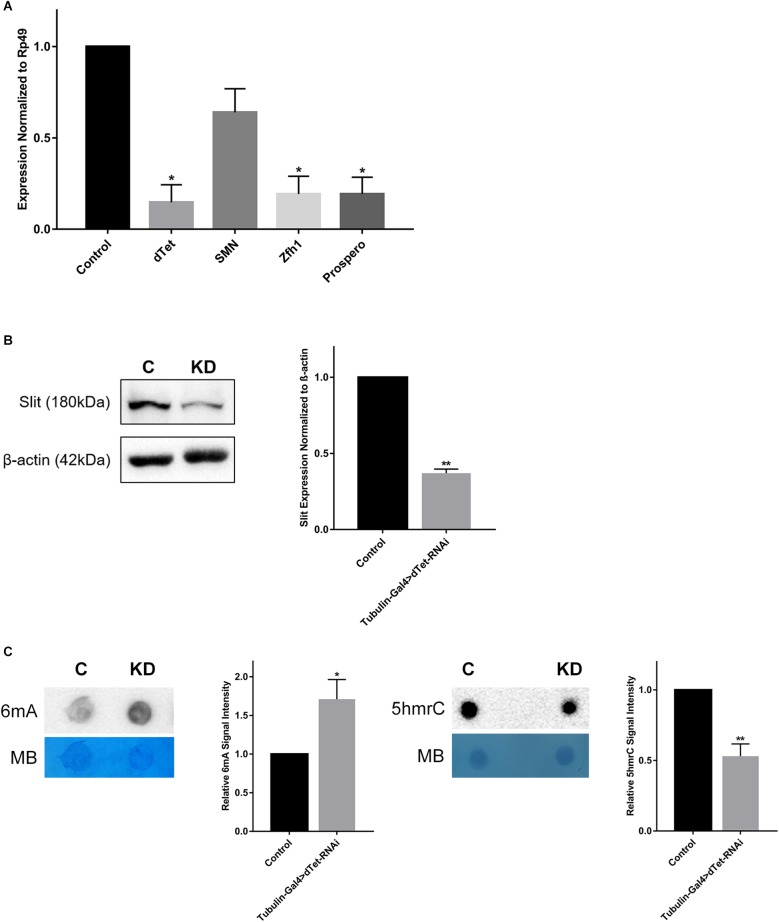
dTet knockdown is associated with downregulation of axon guidance genes and reduced expression of Slit protein. **(A)** qRT-PCR on candidate genes in dTet knockdown larvae showing transcript levels normalized to *Rp49* in Tubulin-Gal4 > w1118. (*n* = 3, 15 larvae per group). Mean is shown with SEM. **(B)** Representative Western blot on protein extracts from larval brains. The 180-kDa band corresponding to the Slit protein is reduced in dTet knockdown brains compared to controls (*p* < 0.05, *n* = 3, 35 brains per group). **(C)** Representative images from dot blot assays on 6mA and 5hmrC abundance in third instar larval brains (*n* = 3). Methylene blue (MB) used as loading control; 600 ng for DNA and 1500 ng for RNA. C: Control (Tubulin-Gal4 > w1118), KD: dTet knockdown (Tubulin-Gal4 > dTet-RNAi). Graphs showing relative abundance of 6mA and 5hmrC marks in knockdown larvae normalized to loading control (*p* < 0.05 and *p* < 0.01, respectively, *n* = 3). Mean is shown with SEM.

dTet regulates the expression of many important genes involved in numerous processes such as developmental, neuronal functions, and axon guidance and was linked to its catalytic activity as a 6mA demethylase (Yao et al., 2018). Therefore, we performed qRT-PCR on three candidate genes – *Prospero*, *Zfh1* (zinc finger homeobox 1), and *Smn* (survival motor neuron) – that are involved in axon guidance. *Prospero* is a transcription factor that plays a critical role in regulating proliferation and differentiation in the developing fly brain (Hassan et al., 1997; Griffiths and Hidalgo, 2004). Accordingly, loss of *Prospero* leads to a disruption in axon guidance processes that are secondary to a disruption in glial and neuronal cell differentiation (Vaessin et al., 1991). *Zfh1* is a transcription factor that is expressed in motor neurons and regulates axon projections in the VNC (Layden et al., 2006). *Smn* is expressed in motor neurons and mutants show developmental and locomotor phenotypes similar to those seen in dTet knockdown larvae (Praveen et al., 2012). *dTet* transcript levels were significantly reduced upon *dTet* knockdown (Figure 4A). While we detected significant downregulation of *Prospero* and *Zfh1* in *dTet* knockdown larvae relative to controls, *Smn* expression was only slightly decreased (Figure 4A). However, the number of early GMCs in third instar larval VNCs that are Prospero-positive was not changed between control and knockdown brains presumably indicating that the changes observed in gene expression are not due to developmental delay (Supplementary Figure 4). Finally, to understand whether secretion of Slit from MG cells is affected in dTet knockdown and whether this is correlated with commissure defects seen in dTet knockout flies, we analyzed levels of Slit protein expression in dTet knockdown larvae by Western blot. Intriguingly, Slit expression was considerably diminished (63%) in dTet knockdown larvae compared to controls (Figure 4B and Supplementary Figure 5). Finally, to determine whether dTet knockdown affected primarily DNA or RNA, we analyzed 6mA and 5hmrC levels using dot blot assays. Both appeared to be affected as 6mA levels were increased and 5hmrC levels were decreased (Figure 4C).

## Discussion

Ten-Eleven Translocation is an important epigenetic regulator that is frequently mutated in cancer including solid tumors. Here, we investigated the role of TET in the model organism *Drosophila melanogaster* focusing on developing brains where dTet is highly expressed. Consistent with previous studies loss of dTet led to survival and locomotor defects (Zhang et al., 2015; Delatte et al., 2016; Wang et al., 2018). We determined that the locomotor dysfunction may be due to the absence of dTet in the midline glia since slit-driven knockdown of *dTet* led to a sharp drop in locomotor performance similar to that seen in ubiquitous knockdown. We hypothesize that the locomotor defect is primarily mediated by the function of midline glia as they appear to be indispensable for proper axon formation in the VNC. To our knowledge, this is the first study to show that midline glia could play a role in locomotor function.

The pathways and circuits underlying locomotion in *Drosophila* have been well-characterized and mapped anatomically. The larval VNC is organized segmentally into abdominal and thoracic sections, with motor axons exiting on both sides to contact peripheral muscles in the body (Clark et al., 2018). Since there are approximately 40 motor neurons per hemineuromere in the VNC, it will be possible to determine the precise neurons that may be involved (Landgraf and Thor, 2006). In addition, a comprehensive screen of genes that are involved in the outgrowth and patterning of motor neuron axons can provide further details on the underlying causes for the disruption seen in the VNC upon *dTet* knockdown.

*dTet* knockdown brains showed a major disruption in the stereotypical axon patterning in the VNC. Proper establishment of axonal connections is dependent on a complex process of attraction and repulsion. Flies lacking Netrin, a chemoattractant, lose all axon commissures (Harris et al., 1996). We did not observe a loss of commissures but rather a mistargeting of commissural axons. Some of these may be motor axons and therefore could explain the observed locomotor phenotype. Interestingly, glia in the optic lobes also play a role in local axon pathfinding (Poeck et al., 2001). Since dTet was also found to be expressed in optic lobe glial cells, we speculate that dTet plays a major role in various types of glial cells that regulate axon guidance.

Both MG-specific and ubiquitous *dTet* knockdown were associated with aberrant commissure crossing, indicating that dTet is at least partially required for MG-based axon guidance. However, since brains from larvae with ubiquitous *dTet* knockdown showed higher severity of the axon commissure defects than MG *dTet* knockdown, there may be other cells that play a role in this phenotype. dTet was present in the majority of neurons in the larval brain and due to the essential interaction between neurons and MG for proper axon formation (Wheeler et al., 2009), the lack of dTet in neuronal cells may contribute to the defect.

Moreover, we identified diminished expression of genes involved in axon guidance. Our findings are in line with a recent paper in which data from an RNA-seq on dTet knockdown in a fly neuronal cell line showed the downregulation of genes involved in axon guidance (Yao et al., 2018) including *Zfh1* and *Prospero*. This was paralleled by an increase in 6mA, which may indicate that the effect of dTet on these genes is mediated by its catalytic function. This is further confirmed by our current study in which we identified an enrichment in the 6mA mark on DNA from larval brains in the absence of dTet. Furthermore, as previously described, we also detect a decrease in 5hmrC, which is the direct product of dTet’s catalytic activity (Delatte et al., 2016). Interestingly, the DNA and RNA marks that dTet catalyzes may result in distinct effects in the developmental program. Whether there is a time- or tissue-dependent alternation between these two marks throughout development remains to be elucidated. Taken together, our findings shed light on fundamental information regarding the role of dTet in the developing brain and point toward an essential role of dTet in midline glia. Further studies are required to decipher the specific underlying mechanisms.

## Author Contributions

JI and SB performed the experiments in this study. MS and FF participated in the design of the study. JI completed the data analysis, and wrote the manuscript. MS supervised the project. WA-K assisted in supervision of the project. FF, WA-K, and MS revised the manuscript. All authors read and approved the final version of the manuscript.

## Conflict of Interest Statement

The authors declare that the research was conducted in the absence of any commercial or financial relationships that could be construed as a potential conflict of interest.
