# Supplementary figures and images for: Drosophila Tet Is Expressed in Midline Glia and Is Required for Proper Axonal Development
Source: Front Cell Neurosci. 2019 Jun 4;13:252. doi: 10.3389/fncel.2019.00252 (PMC6558204; doi:10.3389/fncel.2019.00252)

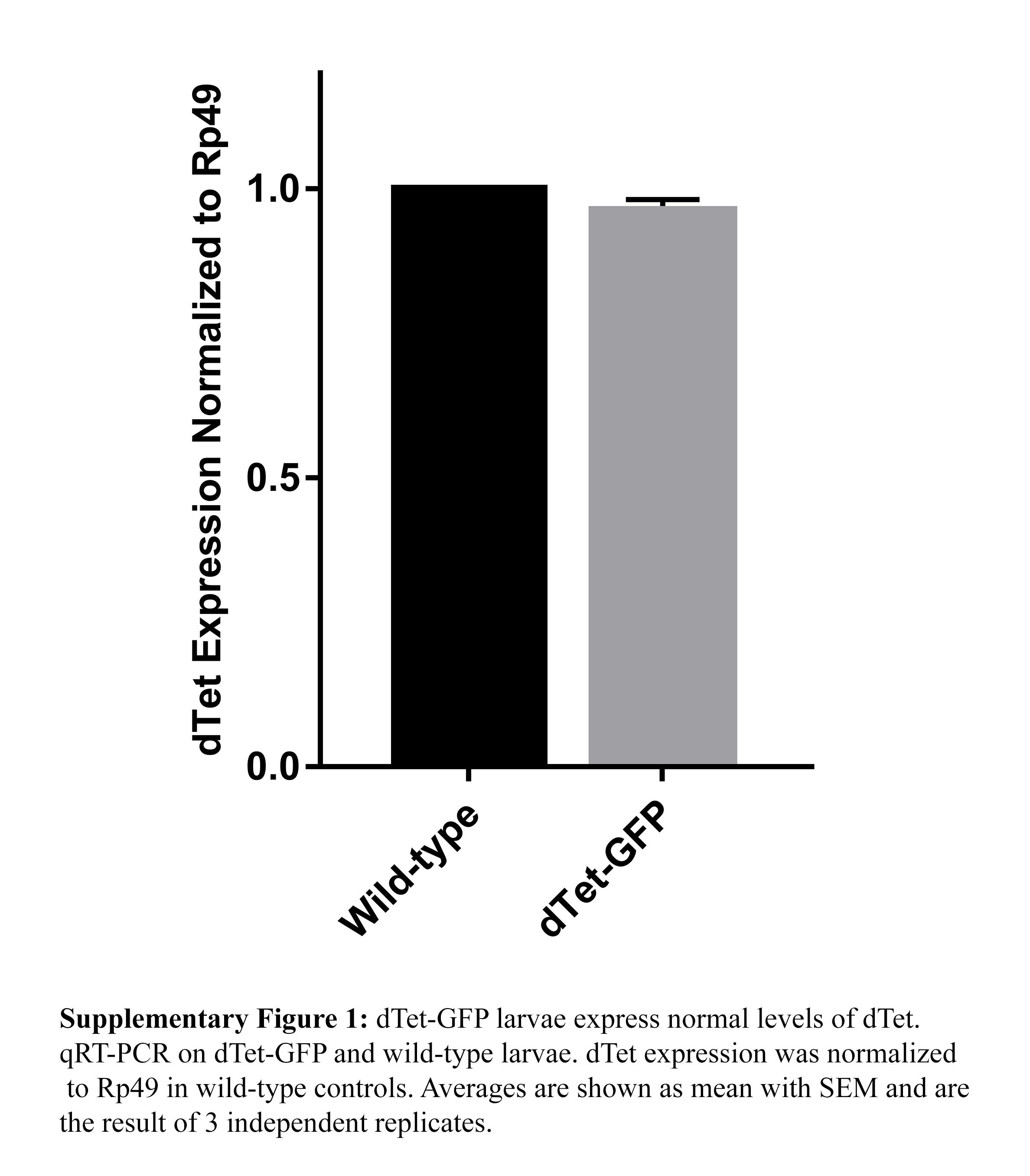

Supplement: Supplementary file 1 [file Image_1.tif]

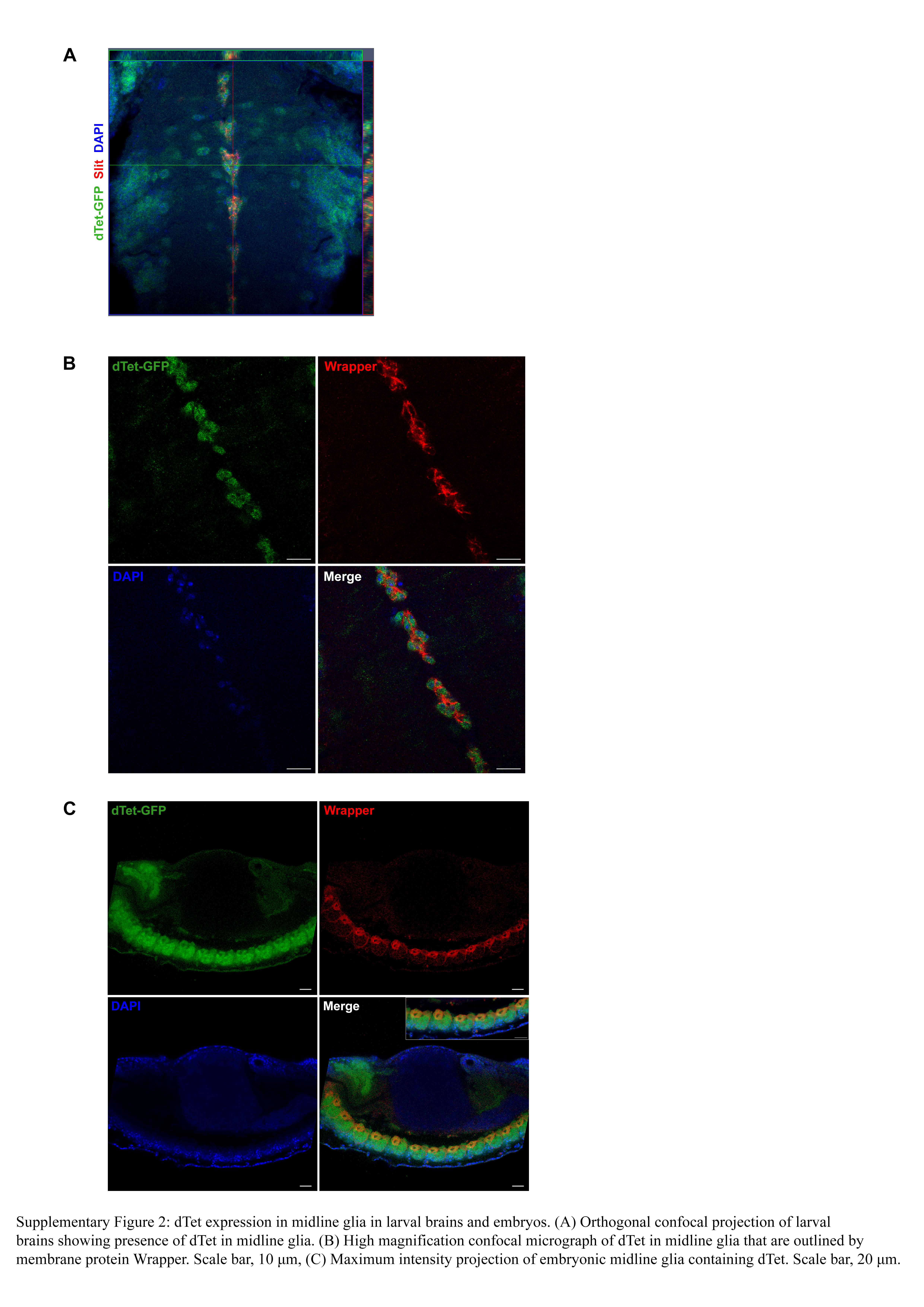

Supplement: Supplementary file 2 [file Image_2.jpg]

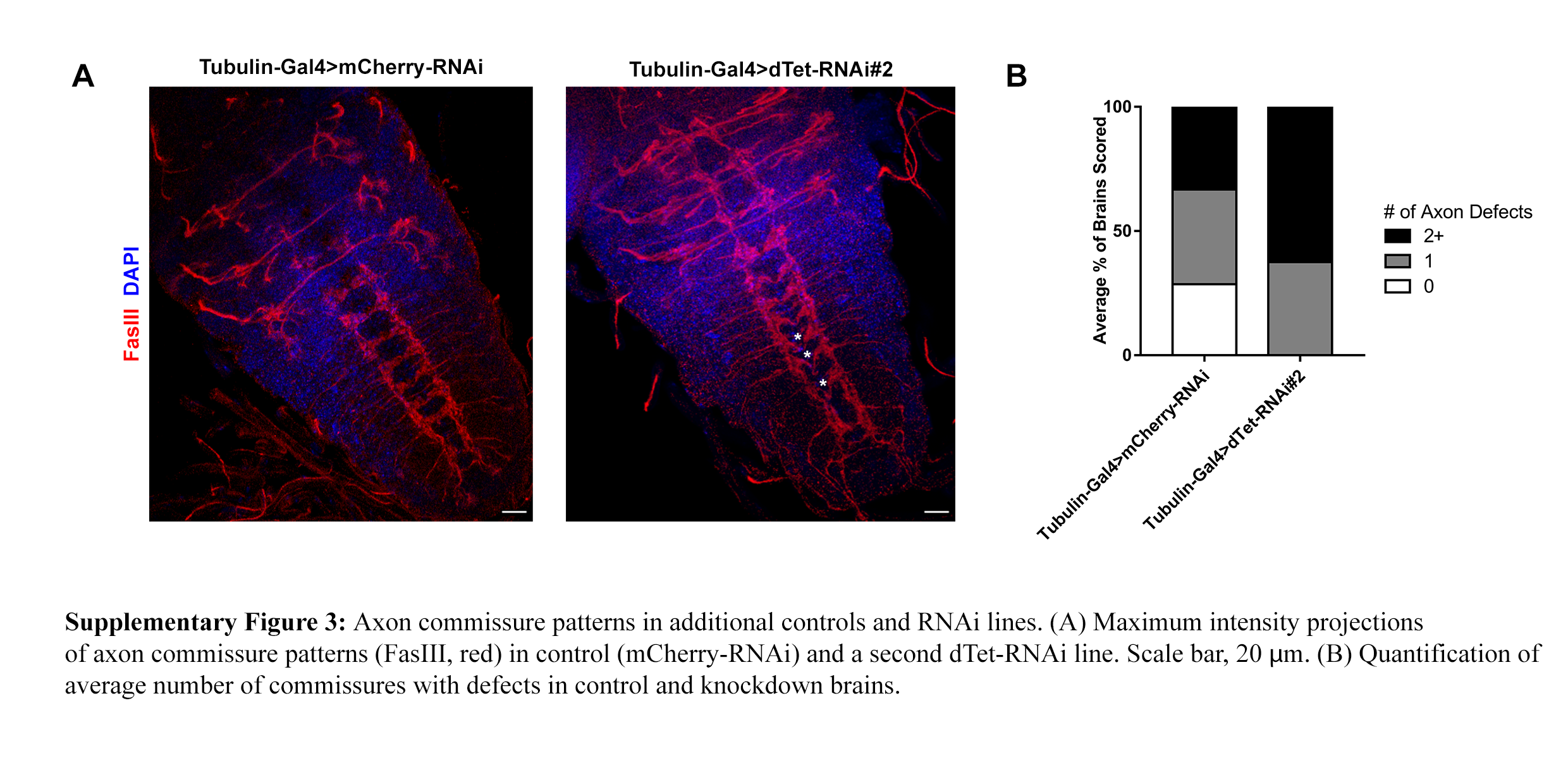

Supplement: Supplementary file 3 [file Image_3.tif]

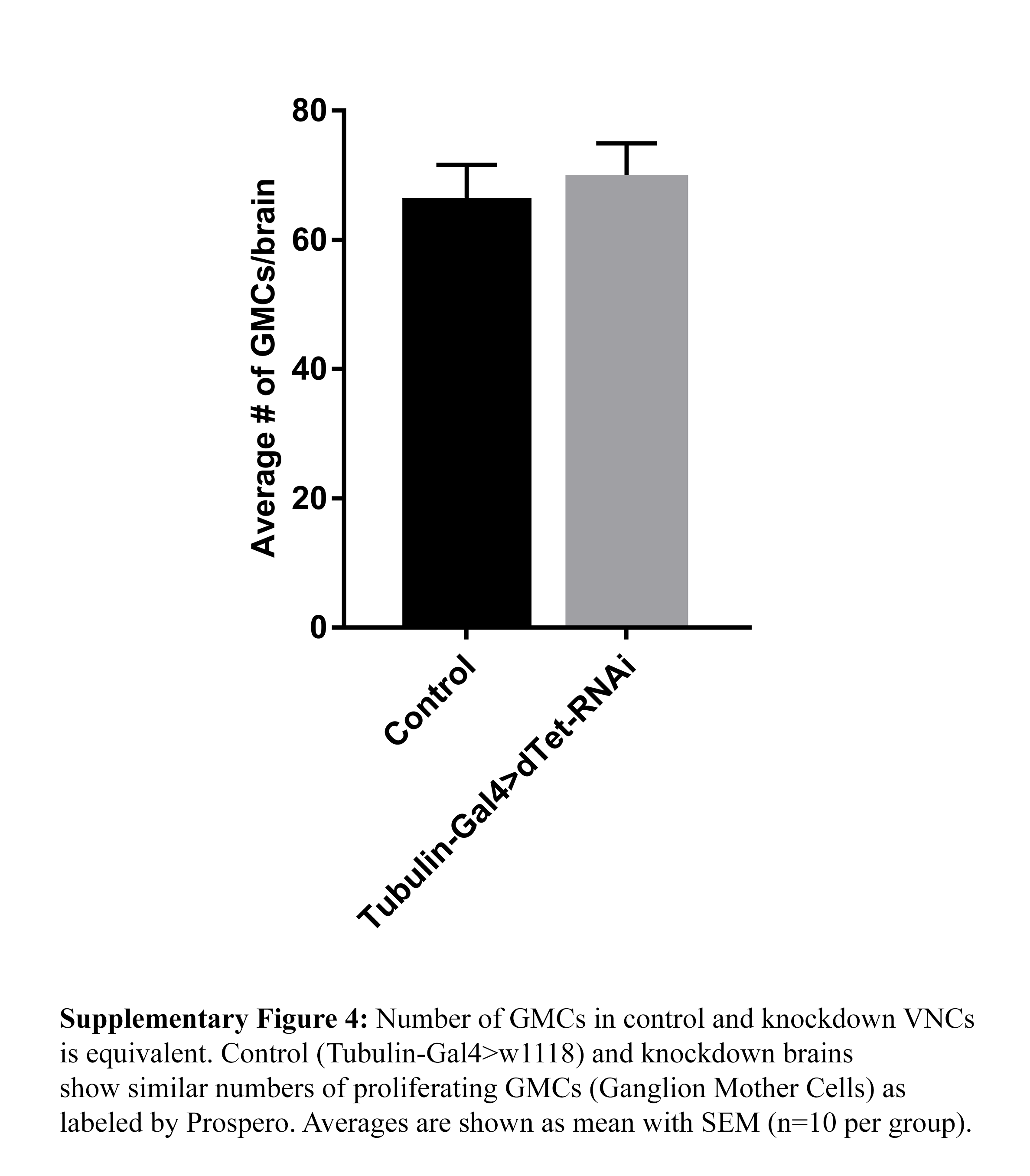

Supplement: Supplementary file 4 [file Image_4.tif]

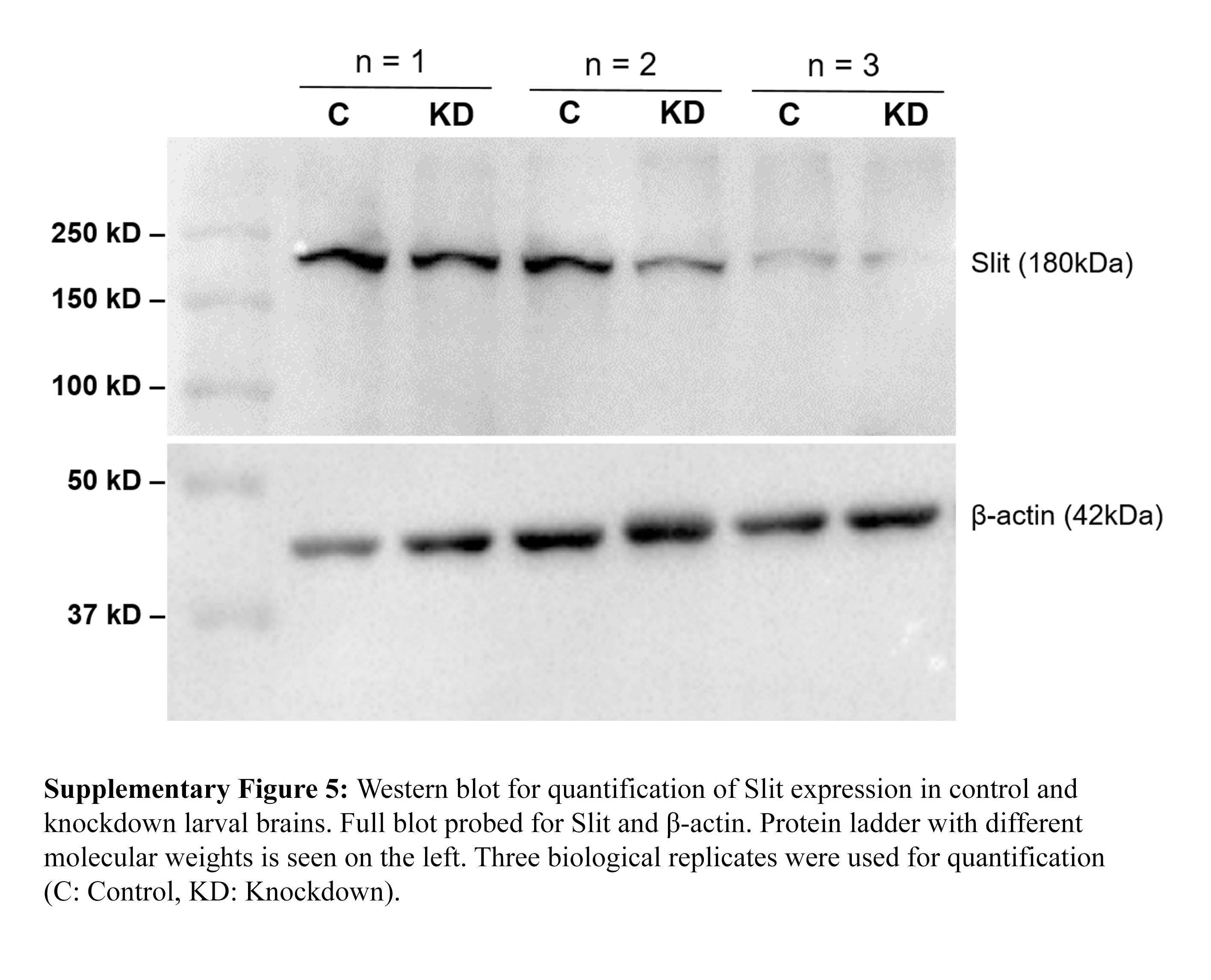

Supplement: Supplementary file 5 [file Image_5.jpg]
