# Supplementary material for: Drosophila Tet Is Expressed in Midline Glia and Is Required for Proper Axonal Development
Source: Front Cell Neurosci. 2019 Jun 4;13:252. doi: 10.3389/fncel.2019.00252 (PMC6558204; doi:10.3389/fncel.2019.00252)
Supplement: Supplementary file 6 [file Table_1.docx]

**Supplementary Table: Primers used in qRT-PCR/Sequencing.**

| **Target** | **Primer Orientation** | **Sequence (5’-3’)** |
| --- | --- | --- |
| *Rp49* | Forward | CGGATCGATATGCTAAGCTGT |
|  | Reverse | GCGCTTGTTCGATCCGTA |
| *dTet** | Forward | AATGCTGGACAAATTTCCCT |
|  | Reverse | CCTTCGATTTGCTACTAGCTC |
| *SMN* | Forward | CCTAAGGCATCCGCTGGTAG |
|  | Reverse | TCTTTCCGCTAGTGGTGCTG |
| *Zfh1* | Forward | GGAAGTTCTCCACATCCGCA |
|  | Reverse | CGCATCTTAAACAGCGCCTC |
| *Prospero* | Forward | CGAGGACGACGACGATGATT |
|  | Reverse | CGCTAATCCGGAGGATGGAC |
| *EGFP*** | Forward | GGATGACGGCACCTACAAGAC |
|  | Reverse | GTGGCTGTTGAAGTTGTACTC |

*Primer sequences obtained from Zhang et al., 2015

**Primer sequences obtained from Venken et al., 2011
